# Causality methods to study the functional connectivity in brain networks: the basal ganglia – thalamus causal interactions

**DOI:** 10.1007/s11682-023-00803-4

**Published:** 2023-10-12

**Authors:** Clara Rodriguez-Sabate, Albano Gonzalez, Juan Carlos Perez-Darias, Ingrid Morales, Miguel Sole-Sabater, Manuel Rodriguez

**Affiliations:** 1 Laboratory of Neurobiology and Experimental Neurology, Department of Physiology, Faculty of Medicine, University of La Laguna, Tenerife, Canary Islands Spain; 2 Center for Networked Biomedical Research in Neurodegenerative Diseases (CIBERNED), Madrid, Spain; 3 Department of Physics, University of La Laguna, Tenerife, Canary Islands Spain; 4 Department of Neurology, La Candelaria University Hospital, Tenerife, Canary Islands Spain

**Keywords:** Neural networks interactions, Functional connectivity, Basal ganglia, Cause/effect relationship

## Abstract

This study uses methods recently developed to study the complex evolution of atmospheric phenomena which have some similarities with the dynamics of the human brain. In both cases, it is possible to record the activity of particular centers (geographic regions or brain nuclei) but not to make an experimental modification of their state. The study of “causality”, which is necessary to understand the dynamics of these complex systems and to develop robust models that can predict their evolution, is hampered by the experimental restrictions imposed by the nature of both systems. The study was performed with data obtained in the thalamus and basal ganglia of awake humans executing different tasks. This work studies the linear, non-linear and more complex relationships of these thalamic centers with the cortex and main BG nuclei, using three complementary techniques: the partial correlation regression method, the Gaussian process regression/distance correlation and a model-free method based on nearest-neighbor that computes the conditional mutual information. These causality methods indicated that the basal ganglia present a different functional relationship with the anterior-ventral (motor), intralaminar and medio-dorsal thalamic centers, and that more than 60% of these thalamus-basal ganglia relationships present a non-linear dynamic (35 of the 57 relationships found). These functional interactions were observed for basal ganglia nuclei with direct structural connections with the thalamus (primary somatosensory and motor cortex, striatum, internal globus pallidum and substantia nigra pars reticulata), but also for basal ganglia without structural connections with the thalamus (external globus pallidum and subthalamic nucleus). The motor tasks induced rapid modifications of the thalamus-basal ganglia interactions. These findings provide new perspectives of the thalamus - BG interactions, many of which may be supported by indirect functional relationships and not by direct excitatory/inhibitory interactions.

## Introduction

Magnetic resonance imaging (MRI) provides information to study the functional connectivity of brain centers in awake humans (functional connectivity MRI; fcMRI). This method uses the fluctuation of the blood oxygenation level dependent (BOLD) signal of two brain centers to establish their functional connectivity (Arthurs & Boniface, 2002; Logothetis, 2002; Logothetis & Wandell, 2004; Raichle, 1998; Raichle & Mintun, 2006). However, the utility of fcMRI methods to group many centers in complex networks and to characterize their internal dynamic is limited (Fox & Raichle, 2007; Goebel et al., 2006; Lee et al., 2011; Rodriguez-Sabate et al., 2019a, b, c). The understanding of the behavior of complex natural networks normally needs the experimental evaluation of cause/effect relationships between their components. In this respect, an event is considered as the cause of another when its intentional activation is frequently followed by the triggering of the second event (Rodriguez-Sabate et al., 2020). This action is normally not possible in the human brain, particularly in neural networks composed of nuclei located deep in the subcortical areas of the brain. Thus, can the BOLD signal fluctuations of the brain centers of a network be used to identify cause/effect relationships and to characterize the behavior of neural networks? The mathematical methods to detect cause/effect relationships in time-series of interacting elements that cannot be experimentally manipulated began during the 1950s-1960s (mainly with the Wiener and Granger studies). These methods have shown an accelerated development in recent years (Barnett et al., 2018; Hillebrand et al., 2016; Korzeniewska et al., 2008; Korzeniewska et al., 2011; Meier et al., 2017; Runge, 2018a; Runge et al., 2019a; Saggioro et al., 2020; Seth et al., 2015; Sugihara et al., 2012), where they have proven to be very suitable for the characterization of climate dynamics (Runge, 2018a; Runge et al., 2019a; Saggioro et al., 2020). Some of these methods have been adapted here to study the cause/effect relationships between basal ganglia, brain centers of a neural network located deep in the human brain and which cannot be experimentally manipulated.

Basal ganglia (BG) are functionally connected with the brain cortex by four cortico-subcortical networks, one of which is the basal ganglia motor circuit (BGmC) (Alexander et al., 1986; Hoover & Strick, 1993; Nambu, 2011). The BGmC transmits information from the primary somatosensory (S1) and motor (M1) cortex to the caudal striatum and subthalamic nucleus (STN), and then to the internal globus pallidum (GPi), external globus pallidum (GPe) and substantia nigra pars reticulata (SNr). Information from the GPe and SNr goes to the anterior-ventral thalamus (motor thalamus; M-Tal) and returns to the S1 and M1 (Alexander & Crutcher, 1990; DeLong, 1990; Obeso et al., 2000). This cortico-subcortical loop is composed of three subcomponents: the direct (M1→Put→SNr/GPi→M-Tal→M1), indirect (M1→Put→GPe→STN→ GPi/SNr→M-Tal→ M1) and hyperdirect (M1→STN→ SNr/GPi→M-Tal→M1) circuits that compete for the functional control of M1 activity (Albin et al., 1989; Alexander et al., 1986; DeLong, 1990; Penney & Young, 1986). These cortico-subcortical loops are normally interacting with other subcortical circuits that do not involve the brain cortex. One of these include the intralaminar (IL-Tal) and mediodorsal thalamus (MD-Tal) thalamic centers (Metzger et al., 2010; Metzger et al., 2013) which receive inputs and send projections to different BG (Benarroch, 2008; Galvan & Smith, 2011; Huerta-Ocampo et al., 2013; Smith et al., 2009; Smith et al., 2004), forming short closed-loop circuits with the direct and indirect pathways (McHaffie et al., 2005; Redgrave et al., 1992). Therefore, the thalamus presents interactions with the BG circuits which are highly complex and whose study with fcMRI methods is particularly challenging.

The present work uses recently introduced mathematical frameworks to identify cause/effect relationships in complex systems which, as is the case of BG, can be recorded but not directly manipulated (e.g. climate evolution) (Barnett et al., 2018; Hillebrand et al., 2016; Meier et al., 2017; Runge, 2018a; Runge et al., 2019a; Saggioro et al., 2020; Seth et al., 2015; Sugihara et al., 2012) (Runge et al., 2019a), to study cause/effect relationships of centers of the thalamus-BGmC neuronal network.

## Materials and methods

### Participants

Twenty-two right-handed volunteers with no history of neurological or mental disease participated in this study (11 males and 11 females between 21–67 years of age; 42.3 ± 9.5 years old). Written informed consent was provided by all participants, all procedures were in accordance with the ethical standard of the 1964 Helsinki declaration, and the study was approved by an institutional review board (Institutional Human Studies Committee of La Laguna University).

### Data collection

The basic experimental procedures were similar to those reported in previous studies (Rodriguez-Sabate et al., 2015, 2017b). Two experimental conditions were used, the resting-task condition with subjects maintaining their body posture and not performing any planned movement and the motor-task condition with subjects performing a repetitive sequence of finger extensions/flexions with the right hand. BOLD-contrast images (4x4x4 mm voxels in-plane resolution; echo-planar imaging with repetition time 1.6s; echo time 21.6 msec; flip angle 90º) were recorded in blocks of 100 volumes in the following sequence: motor block → resting block → motor block → resting block (400 total volumes/subject = 100 volumes x 2 motor-blocks x 2 resting-blocks). fMRI data were co-registered with 3D anatomical images (1x1x1 mm voxel resolution; repetition time 7.6 ms; echo time 1.6 ms; flip angle 12º; 250 x 250 mm field of view; 256x256 sampling matrix). All data sets were normalized to the Talairach space (Table 1 shows the position and size of ROIs).

**Table 1:** Coordinates (Talairach) are shown in mm

		X		Y		Z		Size	
Primary motor cortex		37.2 ± 5.0		-18.3 ± 4.2		47.5 ± 4.9		34.0 ± 10.2	
Primary sensitive cortex		35.6 ± 7.1		-22.3 ± 2.2		50.6 ± 7.2		36.9 ± 8.1	
Caudate		8.7 ± 0.3		11.7 ± 1.1		3.9 ± 1.4		53.3 ± 11.0	
Putamen		26.4 ± 1.4		-4.2 ± 1.0		0.1 ± 0.7		30.4 ± 3.3	
External pallidum		14.4 ± 2.9		-2.0 ± 1.0		3.2 ± 1.0		29.4 ± 3.3	
Internal pallidum		14.2 ± 1.8		-6.3 ± 1.1		-2.4 ± 1.7		27.3 ± 2.3	
Subthalamic nucleus		10.8 ± 1.6		-13.1 ± 2.3		-4.4 ± 2.1		20.8 ± 2.2	
Substantia nigra		7.4 ± 0.7		-18.0 ± 1.2		-8.8 ± 2.2		270.3 ± 47.3	
Intralaminar thalamic nuclei		4.3 ± 1.2		-117.4 ± 1.3		3.7 ± 1.6		52.3 ± 17.1	
Ventral-anterior thalamus		9.2 ± 1.2		-6.4 ± 1.3		7.4 ± 2.2		51.6 ± 11.0	
Medial dorsal thalamus		6.3 ± 1.3		-16.4 ± 1.7		13.6 ± 1.8		61.2 ± 12.6	

The size of the ROIs is shown by the number of their voxels

**Fig. 1:**
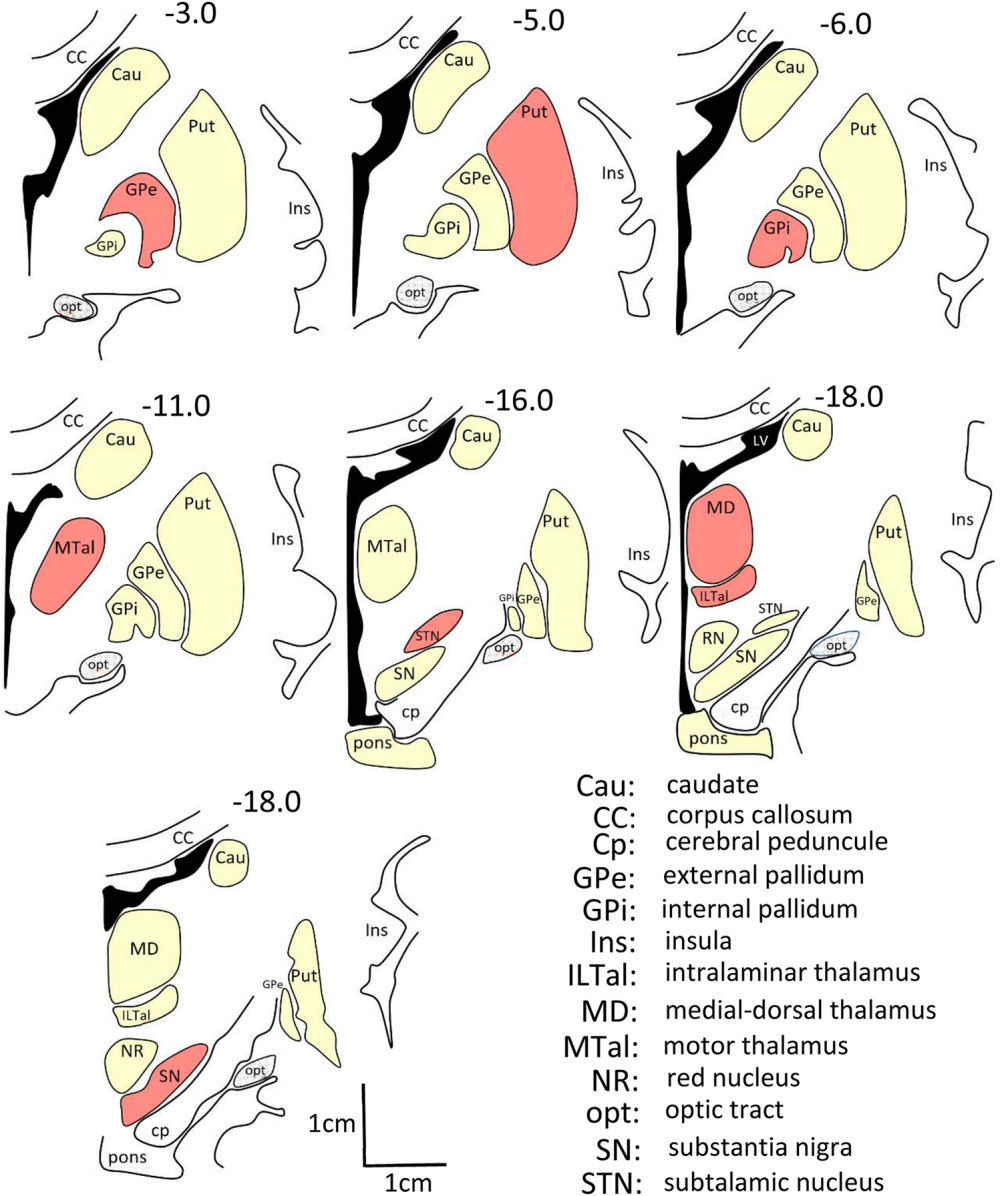
Identification of centers. Time-series used in this study were obtained by averaging (at each time-point) the BOLD-data of the voxels of a region of interest (ROIs) which was manually located in each BG of each subject (T1 normalized 3D-anatomical spaces). The brain position of each BG (red) is shown in coronal slices whose location regarding the optic chiasm is indicated in millimeters (top-right of each figure). To prevent the contamination of data from different centers, ROIs were always located in the central area of each basal ganglia

BOLD time series normally contain coherent fluctuations which are unrelated to neural activity and which originate from residual motion artifacts and physiological signals induced by respiration and cardiac activity. These artifacts may induce an overestimation of functional connectivity strengths. To prevent the confusing effect of artifacts the BOLD-signal of BG were regressed with the BOLD-signals recorded in white matter and brain ventricles (which are sensitive to respiration, cardiac effects, scanner instabilities and other confounding variables..) (Jo et al., 2013b; Power et al., 2014). The BG position may present substantial between-subject differences. Time-series used in this study were obtained by averaging the BOLD-data of voxels located in each of the regions of interest (ROIs) which was manually located in each BG of every subject. Figure 1 shows the brain position of each BG in the T1 weighted templates (normalized 3D-anatomical spaces). To prevent the contamination of data from different centers, ROIs were always located in the central area of each basal ganglia. The resolution of the fMRI (4x4x4 mm) and T1 (1x1x1 mm) images was planned in such a way that one fMRI voxel corresponded with a whole number of T1 voxels (one fMRI voxel containing 64 T1 voxels). T1 and fMRI studies were performed in a single recording session, using the same field of view, and keeping the head attached to the head-coil to prevent movement throughout the T1 and fMRI studies. Thus, there was a marked spatial correspondence between the structural and functional images, and the T1-fMRI correspondence did not need spatial motions during their co-registration of images. Representative fMRI ROIs were obtained from the central region of BG previously identified in T1 images. The ROI size was always big enough to represent the center and small enough to prevent the inclusion of the center boundaries. VOI positions were always verified by independent researchers which confirmed their location inside the corresponding center (not touching the center boundary, surrounding centers or fiber tracts).

fMRI VOIs were individually positioned by using T1 weighted templates normalized 3D-anatomical spaces and using three main indications: 1. coordinates in the Talairach space; 2. the shape of the nucleus; and 3. the anatomical relationship of the nucleus with other structures (external cues) (Fig. 1). Centers were identified in coronal slices located 4–27 mm posterior to the anterior commissure (AC). The optic tract (opt) and internal capsule were used as external cues to identify the GPe, putamen (Put), GPi and MTal. The **GPi** was identified ≈ 6 mm posterior to AC just above the opt. The putamen VOIs were located ≈ 5 mm posterior to AC because the somato-sensorimotor regions project primarily to the posterior putamen (Haber, 2003; Nambu, 2011; Parent, 1990; Selemon & Goldman-Rakic, 1985). **GPe** was located ≈ 3 mm posterior to AC and **MTal** was located ≈ 11 mm posterior to AC. **STN** is a small nucleus whose location was defined according to four external cues, the cerebral peduncle (cp), oculomotor nerve (3), opt, and pons. In this case, coronal images are moved backwards and forwards (10↔18 mm posterior to AC) to identify the slice where the oculomotor nerve is trapped between the pons and cerebral peduncle, and the opt is lateralized. In this slice, the STN was identified 10 mm medial to the opt and above a horizontal line crossing the central point of this tract (discontinuous line), and near the medial boundary of the cerebral peduncle (cp). The STN is surrounded by tracts and other nuclei (SN, zona incerta) and, to prevent the data contamination those of near structures, the STN ROI was small and clearly located within the nucleus boundary. This was not the case of the **SN**. The SN was located between the red nucleus and cp (19–25 mm posterior to AC). The SN pars compacta (SNc) is intermixed with the SN pars reticulata (SNr), and both components cannot be clearly separated in human MRI. Thus, the VOI of the SN included the whole nucleus. The MD and ILTal were identified according to previous studies of Metzger et al (Metzger et al., 2013). The identification of the hand representation in **M1** and **S1** was performed in the precentral and postcentral gyrus according to a previously reported procedure (Rodriguez et al., 2004). The comparison of BOLD data recorded during the resting and motor tasks was used to verify the hand representation in the M1 and S.

### Data preprocessing

The data preprocessing included a slice scan time correction, a 3D motion correction, and a time filter which eliminates frequencies below 0.009 Hz. Studies with images showing a displacement > 0.5mm or a rotation > 0.5degrees were removed. No spatial smoothing was performed. Residual motion artifacts and physiological signals unrelated to neural activity (e.g. respiration, cardiac activity) were removed by regressing the BOLD signals recorded throughout the brain with the mean average of the BOLD signals recorded in white matter and brain ventricles (Jo et al., 2013a; Power et al., 2014).

For each brain nucleus, the time series for all the participants were concatenated to obtain two data sequences, one for the motor case and the other for the resting case. As a first step, data for each subject were normalized around the mean. Then each 100 sample block (motor or resting) was concatenated with the other blocks of the same type, two for each person, for the whole set of participants, obtaining a single time series of 100 volumes x 2 motor/resting blocks x 20 participants = 4000 samples. In order to prevent spurious correlations between series due to block concatenation, the first and last 5 samples of each block were filtered using a gaussian moving average window of size 5, smoothing the transitions between different recordings. Although an effort has been made to normalize the data for all individuals, both in selecting the voxels belonging to each ROI and in the amplitudes of the signals, the different hemodynamic responses and other particular characteristics can cause temporal differences to appear in the individual signals hat complicate the interpretation of causality. However, the use of longer time series increases the robustness of the methods. Further experiments were performed with each of the individual series of 100 time steps and also with the concatenated series for each individual (200 time steps), however, very few causal relationships were obtained. As the number of individuals increased, more consistent results were obtained. Therefore, it was considered that the individual series were different realizations on a normalized individual, assuming the error introduced by the different mentioned properties.

The time series for each nucleus and behavior type (motor/resting), Xi, were joined to create multivariate time series, X, of dimension N (with N being the number of brain nuclei considered for each causal discovery; N = 9). The interactions of the different thalamic centers with BG were studied separately as follows: the M-Tal vs. BG, the IL-Tal vs. BG, and the MD-Tal vs. BG, in all cases the motor and resting tasks were computed separately. Therefore, only the causal relationships between the three thalamic nuclei and the main centers of the BGmC were computed. For each time step **X**_t_ = (X^1^_t_, X^2^_t_, …, X^N^_t_).

### Causality analysis

In this work, a causal network algorithm was used to infer dependencies between the eight centers of the BGmC and each of the thalamic centers. In particular, the PCMCI + method was applied to the multivariate time series for the motor and resting cases (Runge, 2018a; Runge et al., 2019b). This causal discovery method consists of two steps. In the first one, it uses a version of the algorithm proposed by Peter and Clark (PC) (Spirtes & Glymour, 1991) but only to selec t the conditions necessary for the following step, reconstructing the causal parents of each nucleus through iterative conditional independence tests. In this case, the procedure is performed separately for lagged sets and contemporaneous sets. In the second step, the momentary conditional independence (MCI) test is applied, which uses the sets of parents to determine the strength of causal relationships, taking advantage of autocorrelation for orientation identification in contemporary links. This feature is especially important in those cases, such as the present one, where the temporal resolution is too coarse. Specifically, Python package Time Series Graph Based Measures of Information Transfer (TiGraMITe), available at https://github.com/jakobrunge/tigramite.git. was used.

The goal in causal discovery is to estimate the causal parents from time series data. Thus, the relationship between two processes (nuclei signals), **X**^i^ and **X**^j^, must be computed using a particular definition of conditional independence to estimate causal links with statistical reliability. In general, conditional independence of X^i^_t−λ_ and X^j^_t_ given **Z**, denoted by X^i^_t−λ_ ⫫ X^j^_t_ | **Z**, can be expressed in terms of the corresponding conditional probabilities:where X^i^_t−λ_ indicates the value in the time series corresponding to nucleus i at lag λ, and **Z** is a subset of all other processes that potentially influence the relationship between the two processes being tested, i.e., a subset of {X^1^_t_, X^2^_t_, …, X^N^_t_, X^1^_t − 1_, X^2^_t − 1,_ …, X^N^_t−1_, …, X^1^_t − T_, X^2^_t − T,_ …, X^N^_t−T_}, where T is the maximum lag considered. As mentioned, in the case of PCMCI+, the lagged and the contemporaneous sets are treated separately. All those links detected by the algorithm between delayed variables are easily orientated, since the cause corresponds to a previous instant of time, that is, for example X^i^_t−λ_ → X^j^_t_. For the contemporaneous links, two consecutive algorithms are applied, which the author named the collider phase and the rule phase. In them, unshielded triples X^i^_t−τ_ → X^k^_t_ ◦−◦X^j^_t_ (τ > 0) or X^i^_t_◦−◦X^k^_t_ ◦−◦X^j^_t_ (τ = 0) where (X^i^_t−τ_, X^j^_t_ ) are not adjacent, are detected and, if possible, oriented .

TiGraMITe provides several statistical methods to test independence hypotheses, which are typically based on specific assumptions about the underlying dependence between processes, three of which have been used in this study. The first one is based on classical statistics and provides a robust theoretical background. It assumes linear relationships between variables, testing the conditional independence through the corresponding partial correlation (**PC**), ⍴(X^i^_t-λ_,X^j^_t_ | **Z**). Specifically, to perform the conditional independence test, a linear model fit of the centered variables X^i^_t-λ_ and X^j^_t_ as a function of Z is considered and an independent and identically normally distributed observational noise ϵ is assumed (Runge, 2018a):

$$\begin{array}{cc}\mathrm{X}^\mathrm{i}_{\mathrm{t}-\mathrm\lambda}=\mathrm{f}_\mathrm{i}(\mathrm Z)\;+\;\epsilon_\mathrm{i}, &\;\mathrm{X}^\mathrm{j}_\mathrm{t}\;=\;\mathrm{f}_\mathrm{j}(\mathrm Z)\;+\;\epsilon_\mathrm{j}\end{array}$$

Then, the corresponding residuals are calculated from the estimated linear functions (f̂), that will be used in the dependency tests:

$$\begin{array}{cc}{\mathrm r}_{\mathrm i}={\mathrm X^{\mathrm i}}_{\mathrm t-\mathrm\lambda}-{\widehat{\mathrm f}}_{\mathrm i}(\mathrm Z), & \;{\mathrm r}_{\mathrm j}={\mathrm X^{\mathrm i}}_{\mathrm t}-{\widehat{\mathrm f}}_{\mathrm j}\;(\mathrm Z)\end{array}$$

The second conditional independence test does not assume linear relationships as in the previous case, but it uses a non-parametric method based on gaussian process regression and a distance correlation test on the residuals (**GPDC**) (Szekely et al., 2007) to test the dependence, allowing the detection of non-linear dependencies. The kernels used to perform the Gaussian processes regression are based on the addition of a radial basis function kernel and one that simulates a white noise, assuming that the noise of the signal is independently and identically normally-distributed. The last one is the conditional mutual information test based on nearest-neighbor (**CMIknn**) estimator (Runge, 2018a). It is the most general dependency measure, and makes no assumptions about the parametric form of the dependencies by directly estimating the underlying joint density. To this end, a nearest-neighbor conditional mutual information estimator is used, in conjunction with a local permutation scheme proposed by Runge (2018b). The non-dependence on a parameterization improves the estimation of conditional independence in cases where the signals present, for example, multiplicative noise. Usually, both in most causal relationship detection methods and in fMRI signal simulators, additive white noise is assumed for simplicity. However, aside from the thermal noise, which can be modeled by independent homoscedastic Gaussian process, the fMRI noise is generally heteroscedastic, temporally correlated, and nonstationary, with most of the power in the low frequencies.

The non-parametric and model-free methods allow the detection of non-linear relationships in complex systems, but they are based on weaker theoretical results. In all cases, even in the linear one, the statistical significance of conditional independence tests was computed using a block-shuffle permutation test (Mader et al., 2013). This prevents the assumption that the samples are independent and identically distributed, as required by analytic methods, because the time series are usually autocorrelated. Runge (2018a) graphically illustrates the advantages and shortcomings of each of the methods for estimating conditional independence from simulated data, assuming linear and nonlinear relationships with a common driver and using both additive and multiplicative noise. The choice of each of the methods has, therefore, direct consequences on the causal relationships obtained. In the above equations, if Z was a common driver for the variables X^i^_t-λ_ and X^j^_t_, and the dependence of X^i^_t-λ_ on Z and X^j^_t_ on Z were not detected, spurious causality between X^i^_t-λ_ and X^j^_t_ could be inferred. On the other hand, if a relationship between X^i^_t-λ_ and X^j^_t_ actually exists, its detection again depends on the method chosen. For example, if the relationship is not linear and a partial correlation is used, the dependence of these variables could not be established.

In this study, a two-sided significance level of 0.01 and a maximum time lag of T = 2 (3.2 s) were chosen (parent processes that occurred after this time and those with a probability of more than 1% were neglected). T was estimated from the observation of the decay of the unconditional lagged dependencies, retaining those lags for which absolute values are clearly larger than the remainder. In addition, in some experiments longer maximum delay times, up to 5, were tested without finding differences in the results.

As in any applied statistical method, several assumptions must be considered. In this case, the most important assumptions are time-order, causal sufficiency, the causal Markov condition, and faithfulness. Time-order means that causes precede effects. Causal sufficiency assumes that all direct common drivers are in the set of observed time series, in other words, there are no other unobserved processes that directly or indirectly influence any other pair of the studied processes. Causal Markov condition implies that once X^i^_t_ parent values are known, all other variables in the past are not relevant for predicting the value of X^i^_t_. Faithfulness, together with causal Markov condition, guarantees that a measured statistical dependence is due to some, direct or indirect, causal mechanism and, conversely, a measured independence implies that there is no direct causal mechanism.

Although not all these conditions are fully satisfied in the time series studied here, the above methods have multiple advantages to study causal relationships, particularly when compared with other simpler and more commonly used methods. The causal discovery algorithms are designed to obtain as many true causal relationships as possible while controlling the number of false positives, being robust to the influence of common drivers and autocorrelation of the time series (Runge et al., 2014).

## Results

**Fig. 2:**
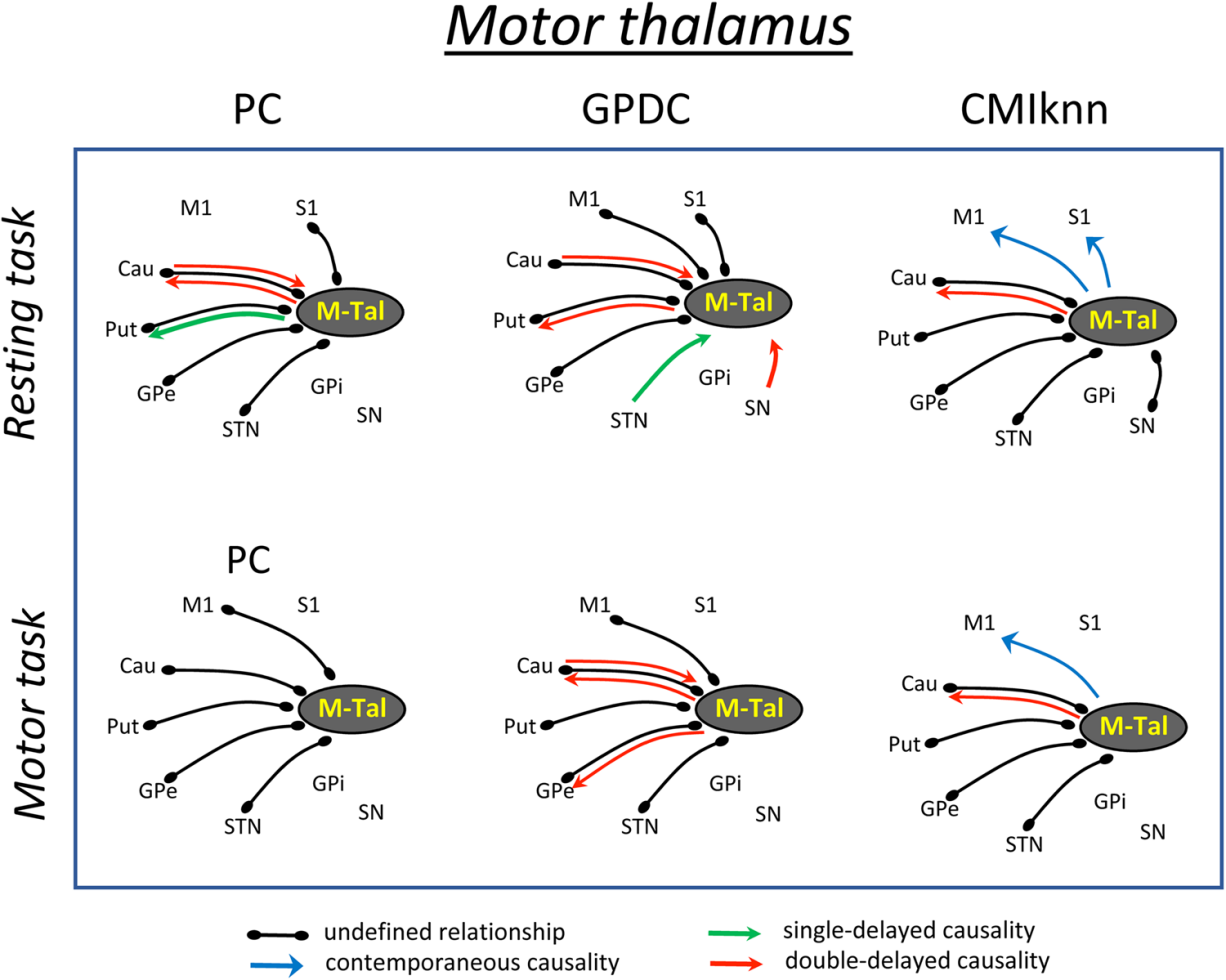
Undefined and causality relationships between the motor thalamus and the basal ganglia motor circuit during the resting-task (top) and the motor-task (bottom). Only the functional connections with statistical value (p < 0.01) are shown. **PC**: partial correlation, **GPDC**: Gaussian process regression and distance correlation, **CMIknn**: conditional mutual information test. **M-Tal**: motor thalamus, **M1**: primary motor cortex, **S1**: primary somato-sensory cortex, **Put**: putamen, **GPe**: external globus pallidum, **STN**: subthalamic nucleus, **GPi**: internal globus pallidum, **SN**: substantia nigra, **Tal**: motor thalamus

**Fig. 3:**
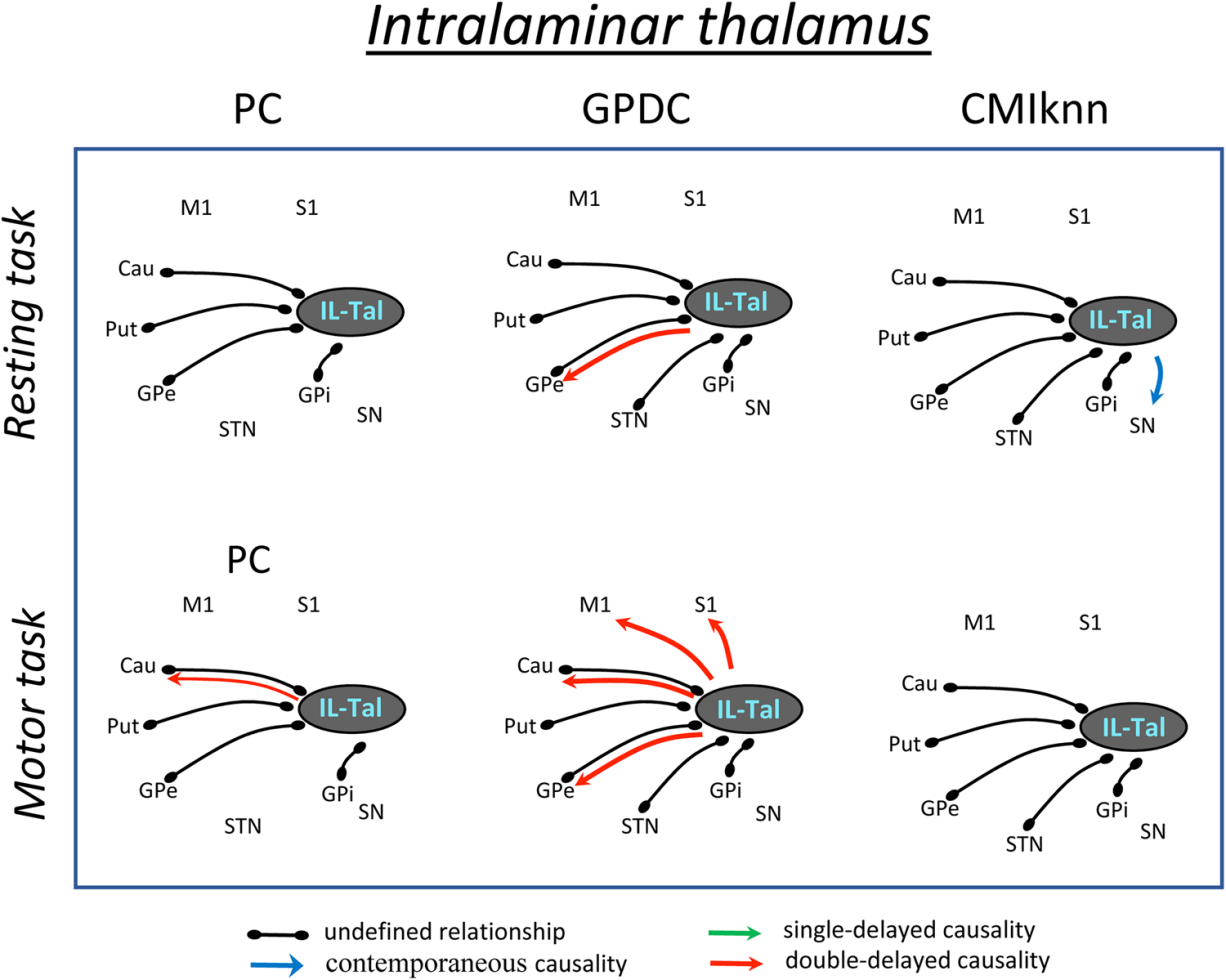
Undefined and causality relationships between the intralaminar thalamus and the basal ganglia motor circuit during the resting-task (top) and the motor-task (bottom). Only the functional connections with statistical value (p < 0.01) are shown. **PC**: partial correlation, **GPDC**: Gaussian process regression and distance correlation, **CMIknn**: conditional mutual information test. **IL-Tal**: intralaminar thalamus, **M1**: primary motor cortex, **S1**: primary somato-sensory cortex, **Put**: putamen, **GPe**: external globus pallidum, **STN**: subthalamic nucleus, **GPi**: internal globus pallidum, **SN**: substantia nigra, **Tal**: motor thalamus

**Fig. 4:**
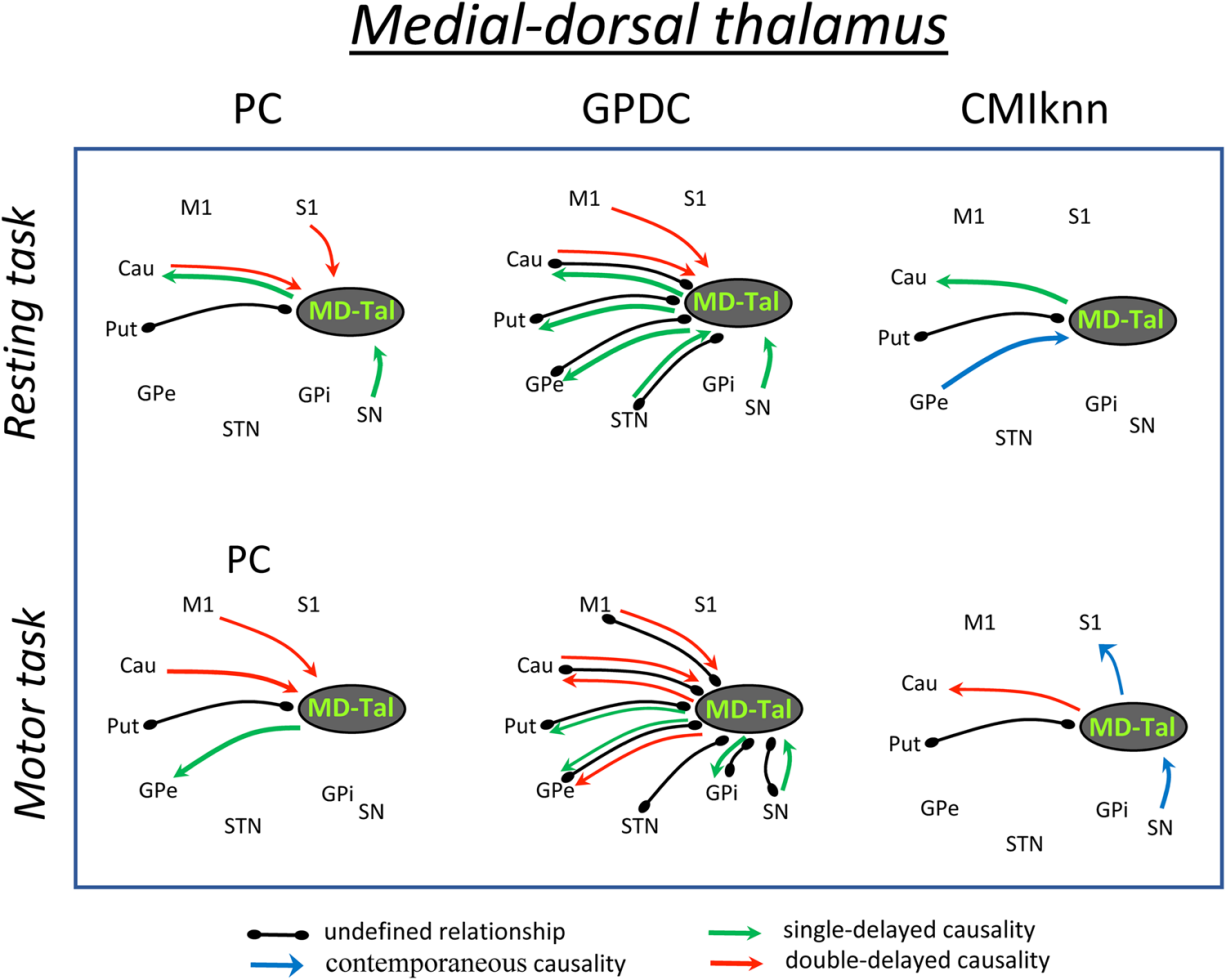
Undefined and causality relationships between the medial-dorsal thalamus and the basal ganglia motor circuit during the resting-task (top) and the motor-task (bottom). Only the functional connections with statistical value (p < 0.01) are shown. **MD-Tal**: Medial-dorsal thalamus, **PC**: partial correlation, **GPDC**: Gaussian process regression and distance correlation, **CMIknn**: conditional mutual information test. **M1**: primary motor cortex, **S1**: primary somato-sensory cortex, **Put**: putamen, **GPe**: external globus pallidum, **STN**: subthalamic nucleus, **GPi**: internal globus pallidum, **SN**: substantia nigra, **Tal**: motor thalamus

**Fig. 5:**
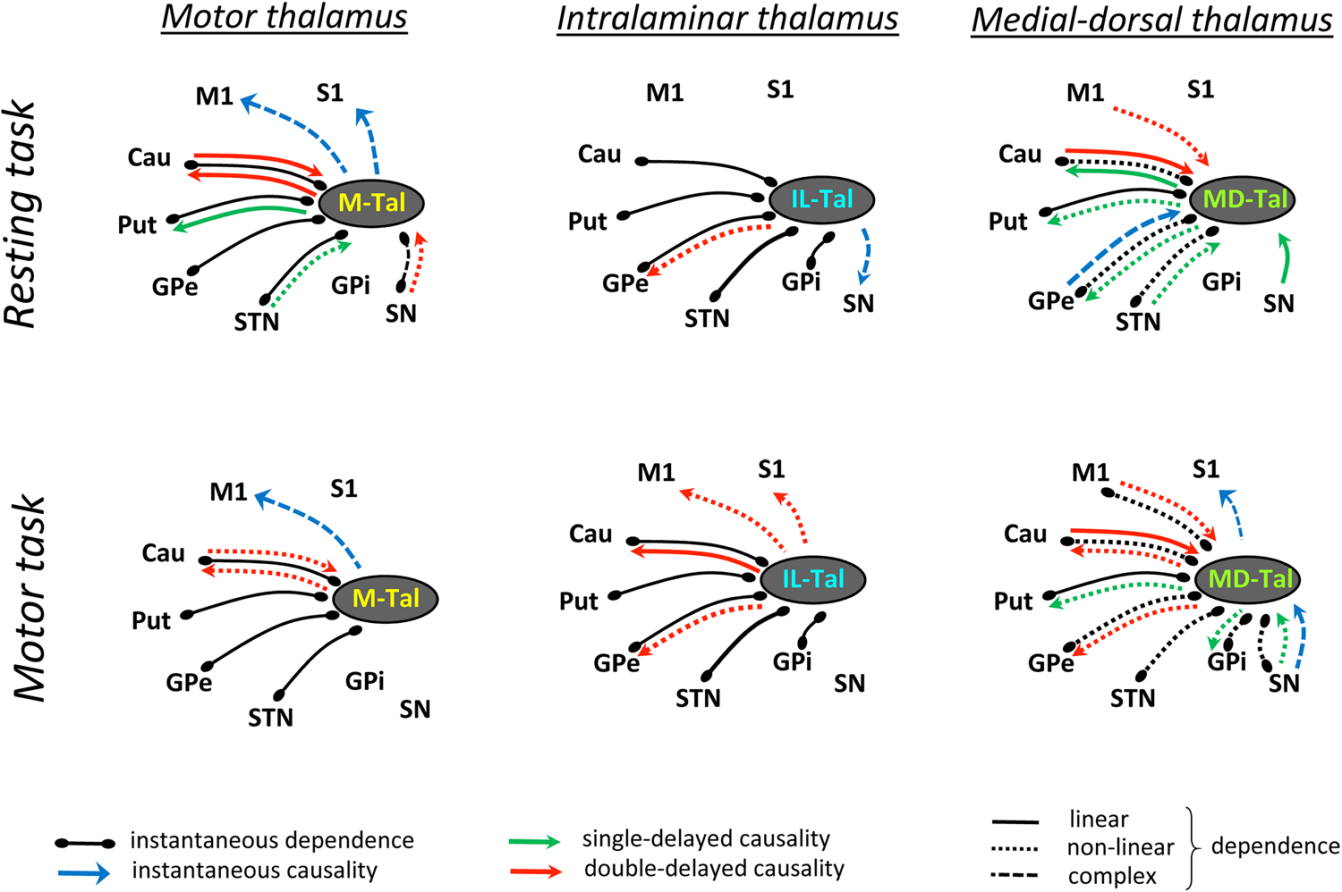
Summary of the thalamic nuclei and the basal ganglia motor circuit during the resting-task (top) and the motor-task (bottom). Only the functional connections with statistical value (p < 0.01) are shown. Linear relationships are shown with continuous lines, non-linear relationships with discontinuous lines, and more complex relationships with dotted lines. **M-Tal**: motor thalamus, **IL-Tal**: intralaminar thalamus, **MD-Tal**: Medial-dorsal thalamus, **PC**: partial correlation, **GPDC**: Gaussian process regression and distance correlation, **CMIknn**: conditional mutual information test. **M1**: primary motor cortex, **S1**: primary somato-sensory cortex, **Put**: putamen, **GPe**: external globus pallidum, **STN**: subthalamic nucleus, **GPi**: internal globus pallidum, **SN**: substantia nigra, **Tal**: motor thalamus

 Functional relationships with statistical values (*p* < 0.01) are shown in Figs. 2, 3, 4, and 5, where the lines indicate undefined relationships that do not allow the identification of the nucleus that causes the functional relationships (causative centers), and the arrows indicate causality relationships from the causative to the response nucleus. A detailed description of each statistical analysis is shown in Table 2. Figure 2 shows the undefined relationships, and the contemporaneous, single-delayed and double-delayed causality found between the motor thalamus (**M-Tal**) and the BGmC nuclei during the resting (top) and motor (bottom) tasks. The functional relationships were identified with the PC (left), GPDC (middle) and CMIknn (right) methods, and only the functional associations with statistical values (*p* < 0.01) are shown here and in the following figures. ***PC*** showed M-Tal undefined relationships with Cau, Put, GPe and STN which were observed during both the resting and motor tasks. In addition, M-Tal showed an undefined relationship with S1 during the resting-task and with M1 during the motor-task. M-Tal induced a single-delayed causality on Put activity and a double-delayed causality/response (***interactive relationship***) interaction with the Cau. These causality relationships were observed during resting and vanished during motion. ***GPDC*** showed an M-Tal undefined relationship with M1, Cau, Put and GPe which was observed during both the resting and motor tasks. In addition, M-Tal showed an undefined relationship with S1 during the resting-task and with STN during the motor-task. M-Tal induced a double-delayed causality on Put (during the motor tasks), Cau (motor-task) and GPe (during the motor-tasks) activity, a single-delayed response to STN activity (resting-task), and a double-delayed response to Cau (resting- and motor-tasks) and SN (resting-task) activity. ***CMIknn*** showed an M-Tal undefined relationship with Cau, Put, GPe and STN (observed during both the resting and motor tasks) and with SN (resting-task). M-Tal induced a double-delayed causality on the Cau activity (resting and motor tasks), and a contemporaneous causality on the M1 (resting and motor tasks) and S1 (resting-task) activity.

**Table 2:** Statistical values of each functional link computed with the partial correlation (**PC**), gaussian process regression and distance correlation (**GPDC**), and conditional mutual information test (**CMIknn**) methods

	I-Method	Lag	M1	S1	Cau	Put	GPe	STN	GPi	SN
M-Tal (resting)	PC	0 1 2 2		-0.306	0.630 -0.529< -0.391*	1.000 -0.148*	0.562	0.205		
	GPDC	0 1 2	0.148	0.148	0.519 0.333<	0.870 0.037*	0.111	0.056<		0.056<
	CMIknn	0 2	0.188*	0.438*	0.375 0.188*	0.688	0.313	0.313		0.375
M-Tal (motor)	PC	0	-0.364		0.791	0.986	0.208	0.205		
	GPDC	0 2 2	0.093		0.815 0.241< 0.167*	0.981	0.407 0.056*	0.222		
	CMIknn	0 2	0.188*		0.313 0.188*	0.500	0.375	0.438		
IL-Tal (resting)	PC	0			0.673	0.983	0.505		0.632	
	GPDC	0 2			0.778	0.778	0.029 0.037*	0.010	0.017	
	CMIknn	0			0.375	0.375	0.375	0.250	0.438	0.250*
IL-Tal (motor)	PC	0 2			0.761 -0.306	0.909	0.558		0.626	
	GPDC	0 2	0.056*	0.056*	0.500 0.204*	0.722	0.667 0.056*	0.130	0.444	
	CMIknn	0			0.375	0.375	0.325	0.313	0.313	
MD-Tal (resting)	PC	0 1 2		0.192<	-0.347* -0.515<	0.777				0.329<
	GPDC	0 1 2	0.074		0.204 0.111* 0.130<	1.000 0.037*	0.093 0.056*	0.056 0.037<		0.185<
	CMIknn	0 1 2	0.250		0.250*	1.000	0.313<			
MD-Tal (motor)	PC	0 1 2			-0.391<	0.724	0.222*			
	GPDC	0 1 2 2	0.074 0.111<		0.241 0.111< 0.111*	0.926 0.037*	0.037 0.019* 0.148*	0.056	0.037 0.074*	0.056 0.074<
	CMIknn	0 2		0.188*	0.250*	0.563				0.188<

Only those relationships with p-values < 0.01 are shown. Quantities without any symbol on the right correspond to relationships in which causality has not been determined. When causality is detected, the * indicates that the thalamic nucleus is the causative center, and the < symbol indicates that the thalamic nucleus is the response. All the statistical values in this table were normalized between 0 and 1, a procedure that facilitates the comparison of the results obtained with the three methods. **M-Tal**: motor thalamus, **IL-Tal**: intralaminar thalamus, **MD-Tal**: Medial-dorsal thalamus, **M1**: primary motor cortex, **S1**: primary somato-sensory cortex, **Put**: putamen, **GPe**: external globus pallidum, **STN**: subthalamic nucleus, **GPi**: internal globus pallidum, **SN**: substantia nigra, **Tal**: motor thalamus

Figure 3 shows the undefined and causality relationships (*p* < 0.01) of the intralaminar thalamus (**IL-Tal**) with the BGmC areas during the resting (top) and motor (bottom) tasks. IL-Tal showed undefined relationships with the Cau, Put, GPe, STN and GPi which were observed with ***PC***, ***GPDC*** and ***CMIknn*** during both the resting and motor tasks. During the resting-task, IL-Tal also induced a double-delayed causality on GPe activity (***GPDC***) and a contemporaneous causality on SN activity (***MCIknn***). During the motor-task, IL-Tal induced a double-delayed causality on Cau activity (**PC** and **GPDC**) and on S1, M1 and GPe activity (***GPDC***).

Figure 4 shows the functional relationships (*p* < 0.01) of the mediodorsal thalamus (**MD-Tal**) with BGmC areas during the resting (top) and motor (bottom) tasks. ***PC*** showed an undefined relationship of the MD-Tal with the Put (resting and motor tasks), and induced a single-delayed causality on Cau (resting-task) and GPe (motor-task) activity. MD-Tal showed a double-delayed response to Cau (resting and motor tasks), M1 (motor tasks) and S1 (resting tasks) activity. ***GPDC*** showed massive interactions of the MD-Tal with all BG and with the M1. MD-Tal showed undefined relationships with the Cau, Put, GPe and STN (resting and motor tasks) and with the M1, GPi and SN (motor-task). MD-Tal induced a single-delayed causality on Cau (resting tasks), and on Put and GPe (resting and motor tasks) activity, and showed a single-delayed response to SN (resting and motor tasks) and STN (resting-task) activity, and a double-delayed response to M1 and Cau (resting and motor tasks) activity. ***CMIknn*** showed undefined relationships between MD-Tal and Put. MD-Tal induced a contemporaneous causality on the S1 (resting-task), and showed a contemporaneous response to the GPe (resting-task) and to the SN (motor-task). In addition, MD-Tal induced a single-delayed causality (resting-task) and a double-delayed causality (motor-task) on the Cau.

Figure 5 simplifies these functional connectivities by grouping the different analytical methods and showing the estimated nature of each relationship according to the method used for its identification. PC identifies linear relationships. GPDC identifies linear and non-linear relationships, and is less sensitive than PC for linear relationships but much more sensitive for non-linear relationships. CMIknn can detect linear, non-linear and more complex relationships, and is less sensitive than PC for linear relationships and than GPDC for non-linear relationships, but is the only method that can detect more complex functional interactions (e.g. chaotic interactions with phase-transitions). In addition, the combination of the CMIknn method (for detecting independence between nodes) and the PCMCI + algorithm was able to detect causality between centers whose BOLD-signal fluctuations were synchronized in the same time-intervals (Runge, 2018a). Taking these facts into account, Fig. 5 shows the linear, non-linear and complex relationships between the three thalamic nuclei studied and the BGmC nuclei.

The **M-Tal** showed four undefined linear relationships (with the Cau, Put, GPe and STN) which did not change with the task (**permanent relationships**), and an undefined complex relationship with the SN, observed during the resting-task but not during the motor-task. The M-Tal induced a contemporaneous complex causality on M1 (resting and motor tasks) and S1 (resting-task) activity and a single-delayed linear causality on Put activity during the resting-task which was detected as a non-linear causality during the motor-task. The M-Tal showed a single-delayed non-linear response to the STN and a double-delayed non-linear response to the SN during the resting-task which vanished during the motor-task. Finally, the M-Tal showed a double-delayed linear interactive relationship with the Cau during the resting-task which became a non-linear interaction during the motor-task.

The **IL-Tal** showed five undefined linear relationships (with the Cau, Put, GPe, STN and GPi), and induced a non-linear double-delayed causality action on GPe activity which did not change with the task. The resting-task produced a contemporaneous complex causality of the IL-Tal on SN activity which was not observed during the motor-task. On the other hand, the motor-task produced a double-delayed non-linear causality of IL-Tal activity on M1 and S1 activity which was not observed during the resting-task.

Most **MD-Tal** interactions with the BGmC centers were of a non-linear nature, even those rapid actions classified as undefined relationships. Although non-linear relationships should also be detected by CMIknn-based methods, they were only detected by those based on GPDC. The MD-Tal showed an undefined linear relationship with Put and undefined non-linear relationships with the Cau, GPe and STN during the resting tasks. These relationships persisted during the motor-task. In addition, the motor-task induced undefined non-linear relationships with the M1 and GPi which were not observed during the resting-task. The MD-Tal induced a non-linear causality action on Put (single-delayed) and GPe (double-delayed) activity during the resting-task, and a linear causality on the Cau (single-delayed) during both tasks (although the single-delayed linear action on Cau observed during the resting-task became a double-delayed non-linear action during the motor-task). MD-Tal induced a contemporaneous complex causality on S1 activity during the motor-task but not during the resting-task. On the other hand, the MD-Tal showed a response to GPe (complex contemporaneous causality), and STN (non-linear single-delayed causality) activity during the resting-task but not during the motor-task. Finally, the MD-Tal worked as response center for the SN, showing a complex contemporaneous response during the motor-task and a single-delayed non-linear response during both the resting and the motor tasks.

## Discussion

Present data show that the analysis of fcMRI data with the causality methods is a useful procedure to advance the understanding of the neuronal networks of human BG. The combination of three independent statistical procedures provided an exhaustive (identifying the functional connectivity regardless of its linear, non-linear or complex nature) and selective (avoiding the spurious relationship generated by the closed-loop arrangement of BG) view of the functional connectivity of the thalamus with the BGmC. Causality relationships were observed in a portion of the functional connectivity, showing the nature (linear, non-linear or complex), the time-dynamic (contemporaneous, single-delayed and double-delayed) and the causative/response centers of each functional relationship. The causality relationships changed with the task, providing a new view of the thalamic action on the BGmC dynamics in the human brain.

### Advantages and disadvantages of present methods

The identification of causes and effects is one of the key facts in the development of experimental sciences. It is generally considered that a fact X is the cause of a fact Y when the repeated manipulation of X has the same effect on Y (“*experimental causality*”). This direct experimental manipulation can rarely be performed in the case of the human brain, particularly in the case of the BG which are located deep below the brain cortex. Present methods used the relative fluctuation of the different BG (BOLD time-series) to estimate the cause/effect relationships involved in the functional interaction of their nuclei. This is a “*statistical causality*” which identifies causation when the probability of X→Y transitions is higher than expected at random. This cause/effect relationship is more easily identified when the X (cause) and Y (effect) are found in successive time-windows, but when they appear in the same time-window (*simultaneity window*) the time lag between X and Y cannot be used to identify the cause and the effect in this statistical association. The BOLD-signal had a time-resolution of 1.6 sec (simultaneity window in this study), and when the phase-shift of BOLD-waves of two nuclei is less than the simultaneity window the statistical causality (**causation**) of their functional relationships cannot be established by the time precedence. However, a new procedure has recently been introduced to estimate cause/effect relationships even in fluctuations with a phase-shift shorter than the simultaneity window (Runge, 2018a, b; Runge et al., 2019a; Saggioro et al., 2020). Here, these methods identified a number of cause/effect relationships between the thalamic and BG nuclei, some of which were found using simultaneous BOLD-fluctuations (contemporaneous causality) and others using non-simultaneous BOLD-fluctuations (single-delayed and double-delayed causality). Conceptually, the causality studied here corresponds to the bivariate Granger causality, bivariate transfer entropy, conditional mutual information and phase transfer entropy computed with other methods. Contemporaneous causality could not be identified in all the simultaneous BOLD-fluctuations with statistical value (undefined relationships), a methodological limitation that future studies could overcome with new analytical methods or using fcMRI recordings with a higher time-resolution.

Another limitation of present methods is caused by some of the physiological characteristics of the BG. These methods require a number of preconditions (stationarity, causal sufficiency, faithfulness, etc.) that cannot always be verified in brain studies. Although special precautions were taken here to prevent artefactual interactions and spurious causalities (e.g. non-parametric significance tests, long time-series, etc.), misidentifications cannot be completely ruled out (Runge, 2018a). Present methods can identify individual interactions between two centers but not multiple simultaneous interactions between the different centers of the same network (functional multinuclear ensembles), which is another limitation of the present study. The independent component analysis (Damoiseaux et al., 2006; Fox & Raichle, 2007; Goebel et al., 2006) and data-driven sparse GLM (Lee et al., 2011; Su et al., 2016) can work with multiple regions at the same time, but they mainly use linear interactions and may be not sensitive to some of the non-linear relationships previously observed in the BG (Marceglia et al., 2006; Rodriguez-Sabate et al., 2017a; Rodriguez et al., 2003a, b, c; Schroll & Hamker, 2013), and which in the present study were found in a high percentage of the thalamus-BGmC relationships. Some new multifactorial methods recently introduced to study the interaction of multiple brain regions may work with non-linear signals, but they do not provide an identification of BG interactions as exhaustive as the present method does, and they do not identify causal relationships (Rodriguez-Sabate et al., 2020; Rodriguez-Sabate et al., 2017a). Present methods provide an exhaustive identification of the functional relationships between the thalamus nuclei and the main centers of the BGmC, most of which showed non-linear dynamics and cause/effect relationships.

The joint application of present analytical methods offers an additional advantage, it provides information about the basic characteristics of the functional relationships. The most sensitive method for linear relationships is the PC. GPDC identifies both linear and non-linear relationships but it is more sensitive for non-linear and less sensitive for linear relationships than the PC. Therefore, the functional relationships have been classified as linear relationships when they were detected by PC, and as non-linear relationships when they were detected by GPDC but not by PC. CMIknn identifies linear, non-linear and more complex functional relationships. This technique is much less sensitive for detecting linear and non-linear relationships and much more time-demanding than the other two methods, but it can identify complex relationships undetectable by the other methods. Thus, the functional relationships not detected by PC and GPDC were identified as complex relationships by CMIknn. The integrated application of the three methods proved to be useful to identify functional BG interactions not observed by other methods, reducing the possibility of incorporating spurious causality into the BG model.

### Causality and thalamus-BGmC structural connectivity

Thalamic nuclei are directly involved in the segregation of the information processed by the BGmC, with each thalamic nucleus showing particular structural connections and different physiological functions. The **M-Tal** receives projections from the GPi and SNr and sends projections to the motor cortex, thus closing the three cortico-subcortical loops of BG, the direct, indirect and hyperdirect loops (Levy et al., 1997; Parent & Hazrati, 1995; Sherman, 2016). A significant portion of the M-Tal neurons also project to the striatum where they may interact with striatal inputs coming from the motor cortex (Haber & McFarland, 2001; McFarland & Haber, 2000, 2001). The **IL-Tal** receives massive projections from the GPi and SNr (together with those coming from the superior colliculus, pedunculopontine nucleus, locus coeruleous, amygdala and other nuclei) (Groenewegen & Berendse, 1994; Sidibe et al., 1997, 2002; Smith et al., 2004), and sends projections to the caudate and Put (together with those going to the motor cortex and to different subcortical areas such as the nucleus accumbens) (Berendse & Groenewegen, 1991; Mandelbaum et al., 2019; Parent & Parent, 2005; Smith et al., 2004). IL-Tal neurons are involved in the cortico-subcortical loops of BG by receiving collaterals of the axons of the GPi/SN neurons that project to the M-Tal and by modulating the striatal action of the cortico-striatal projections (Parent & Hazrati, 1995; Sidibe et al., 1997, 2002). In addition, the IL-Tal generates different subcortical BG loops (e.g. the IL-Tal → ***Put*** → GPi → IL-Tal motor loop, the IL-Tal → ***Cau*** → SNr → IL-Tal associative loop and the IL-Tal → ***accumbens*** → GPi → IL-Tal limbic loop) (Galvan & Smith, 2011; Sidibe et al., 1997, 2002; Smith et al., 2009, 2004). The **MD-Tal** receives inputs from the GPi and SNr and sends outputs to the striatum (Ilinsky et al., 1985; Percheron et al., 1996), although most of its projections go to the prefrontal cortex (Delevich et al., 2015; Heidbreder & Groenewegen, 2003). In addition to these multicenter pathways, the M-Tal, IL-Tal and MD-Tal present reciprocal modulatory interactions with the brain cortex (glutamatergic neurons of these thalamic nuclei innervate glutamatergic neurons of the brain cortex that project to the glutamatergic neurons of the thalamus) (Harris & Shepherd, 2015; Jeong et al., 2016; Lusk et al., 2020; Mandelbaum et al., 2019; Sherman, 2016).

Taken together, all these pathways form a complex network where the information may flux by different routes at the same time and may be continuously recirculating by feed-back reentrant connections. These thalamus-BG networks may use information arriving from different sources to perform different functions (Galvan & Smith, 2011; Haber & Calzavara, 2009; Kimura et al., 2004; McHaffie et al., 2005; Rodriguez-Sabate et al., 2015). This complex structural organization, the reentrant wiring of the BG, and the non-linear (or complex) dynamics previously reported in the BG (Marceglia et al., 2006; Rodriguez-Sabate, 2017b; Rodriguez et al., 2003a, b, c; Schroll & Hamker, 2013) and observed here in many of the thalamus-BG relationships make the understanding of the thalamus-BGmC interaction a challenging task. No particular physiological functions have been identified in each of the thalamus-BGmC networks at the moment, and present data cannot do that. However, present data provide an extensive list of functional interactions between the thalamic nucleus and the main nuclei the BGmC, showing cause/effect relationships in most cases.

### The functional connectivity of the thalamus and BGmC according to the causality methods

The causality methods indicated four key facts: **1.** BGmC nuclei present a different functional relationship with the M-Tal, IL-Tal and MD-Tal; **2.** more than 60% of these thalamus-BGmC relationships showed non-linear or complex dynamics (35 of the 57 relationships found); **3.** the motor tasks induced rapid modifications of the thalamus-BG interactions. **4**. the thalamic nuclei present functional relationships with BGmC nuclei that have direct structural connections with the thalamus (M1, S1, Cau, Put, GPi and SNr), but also with other BG nuclei that do have these connections (GPe, STN). These findings provide new perspectives of the thalamus - BG interactions, many of which may be supported by indirect functional relationships and not by direct excitatory/inhibitory interactions.

The dynamics of the thalamus-BG relationships have been mainly based on the excitatory/inhibitory interactivity of their nuclei, with each nucleus producing a local action on the next nucleus of the BG cortico-subcortical loop, and with the global dynamic of the BG being the result of these local interactions. Present data suggest that each thalamic nucleus can modulate the activity of most BGmC nuclei, even when they do not have direct structural connections. Thus, the thalamic action on BGmC nuclei may be supported by direct or by indirect pathways (e.g. the **IL-Tal** can influence **GPe** activity by different routes including ***IL-Tal***→Put→***GPe***, ***IL-Tal***→Cau→***GPe***, and ***IL-Tal***→M1→Put→***GPe***), with both actions being performed in time-intervals shorter than 100–200 msec. These rapid actions may be at the basis of the undefined relationships or of the contemporaneous causality observed here. On the other hand, the delayed causations require temporal latencies greater than 1600–3200 msec, which suggests that they involve more indirect pathways (e.g. thalamic projections to the prefrontal cortex or the amygdala), or they require many turns of one or several closed-loop networks (e.g. ***IL-Tal***→Put→***GPe***→STN→GPi→***IL-Tal***, ***IL-Tal***→Cau→***GPe***→ STN→GPi→***IL-Tal***, ***IL-Tal***→M1→Put→***GPe***→STN→GPi →***IL-Tal***). The reentrant signaling has been proposed as a mechanism to facilitate the diffusion of information across the cerebral cortex and to facilitate the functional link of cortical areas without direct structural connections (Edelman & Gally, 2013). A key characteristic of BG networks is their circular arrangement, which may be particularly suitable for the reentrant signaling. In this case, the thalamus-BG delayed causality could be the result of the recirculation of information, and several turns of thalamus-BG loops would be necessary for the delayed functional synchronization observed here.

### Influence of the motor tasks on the thalamus-BGmC functional connectivity

**Fig. 6:**
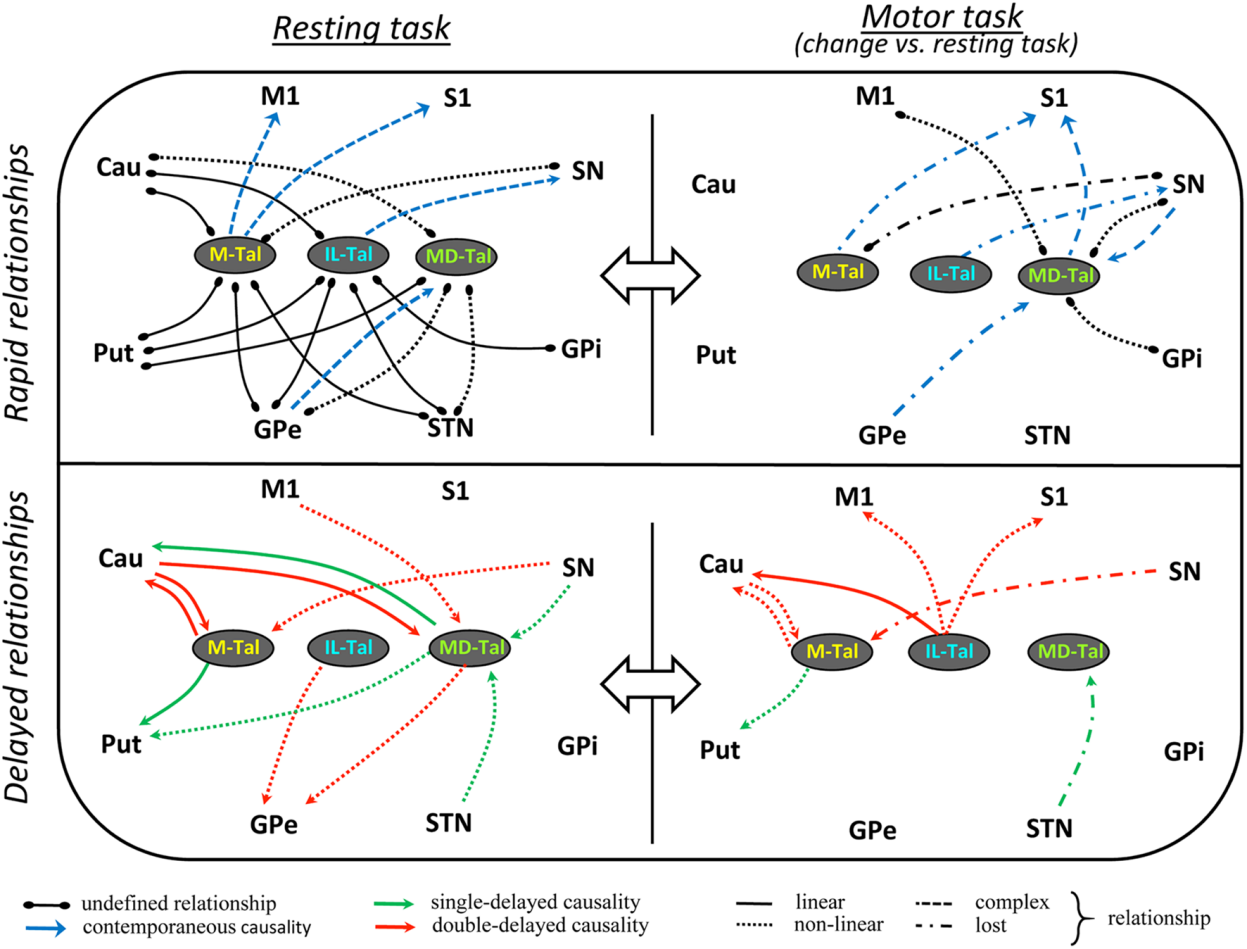
Summary of the rapid (top) and delayed (bottom) functional reconfigurations of the thalamus - basal ganglia interactions induced by the resting-task (left) and motor-task (right). Linear relationships are shown with continuous lines, non-linear relationships with discontinuous lines, and more complex relationships with dotted lines. **PC**: partial correlation, **GPDC**: Gaussian process regression and distance correlation, **CMIknn**: conditional mutual information test (based on nearest-neighbor). **M-Tal**: motor thalamus, **IL-Tal**: intralaminar thalamus, **MD-Tal**: Medial-dorsal thalamus, **M1**: primary motor cortex, **S1**: primary somato-sensory cortex, **Put**: putamen, **GPe**: external globus pallidum, **STN**: subthalamic nucleus, **GPi**: internal globus pallidum, **SN**: substantia nigra, **Tal**: motor thalamus

An interesting finding was the rapid reconfiguration of the functional connectivity of the thalamus induced by the motor task. Figure 6 shows a summary of the reconfiguration of the rapid (top) and delayed (bottom) relationships induced by the motor-task (right side) regarding the resting-task (left-side). In order to simplify the review of results, only the changes induced by the motor-task (vs. the resting-task) are shown in this figure.

The *undefined relationships* observed during the resting-task showed a preponderance of linear connectivity in the M-Tal and IL-Tal (with the Cau, Put, GPe and STN but not with the SN), and of non-linear connectivity in the MD-Tal (with the Cau, GPe and STN but not with the Put). The fact that all the undefined M-Tal and IL-Tal relationships found during the resting-tasks persisted during the motor-task (except the M-Tal vs. SN), and that no new undefined relationships appeared with the motor-task (except the MD-Tal vs. M1), suggest that the rapid connectivity is involved in the preservation of basic functions of BG which could be working in any physiological condition. Some of the rapid functional connections of the M-Tal and IL-Tal showed a causal relationship (*contemporaneous causality*). The M-Tal displayed a contemporaneous causality that modulated the activity of the M1 and S1, and which could be involved in the BG functions performed during resting (the M1 and S1 modulating the muscle tone and body posture) (Mellone et al., 2016; Wright et al., 2007) or during the motor activity (the M1 executing voluntary actions). The **M-Tal** showed undefined rapid relationships with many BGmC areas which are probably supported by the direct structural connections of these areas (Ilinsky et al., 1985; Percheron et al., 1996), and which may be involved in the BG functions performed during the resting (the Cau, Put, GPe and STN) and the motor (the GPi, SN and M1) activity.

The *delayed causality* between the thalamic and BGmC nuclei also changed with the motor-tasks (Fig. 5 bottom). The **M-Tal** showed a double-delayed interactive relationship with the Cau and induced a single-delayed causality on Put activity during the resting-task. These were linear causalities which changed to non-linear causalities (Cau) or vanished (Put) during the motor-task. The **IL-Tal** induced a double-delayed causality on the GPe (non-linear) during the resting task that persisted during the motor task, which was then accompanied by a double-delayed causality on the Cau, M1 and S1 (non-linear). The **MD-Tal** showed complex causality (Cau, Put and GPe) and response (Cau, STN, SN, M1) relationships during the resting-task which did not change with the motor-task (except the loss of the STN→MD-Tal causality).

In summary, present data show that the motor tasks induce a broad action on the functional relationships of the thalamus and BGmC, inhibiting some interactions and activating others, and modifying the time-latency (rapid vs. delayed) and dynamics (linear, non-linear and complex) of different interactions. Future studies using other behavioral tests, faster fcMRI methods and new mathematical algorithms may help to identify the structural substrate and the physiological function of these functional interactions. These studies will need the inclusion of new brain areas (e.g. premotor cortex) (Delevich et al., 2015; Heidbreder & Groenewegen, 2003), new BG loops (e.g. prefrontal cortico-subcortical loop) and particular motor functions (e.g. selection and timing of motor patterns) (Hunt & Aggleton, 1998; Lusk et al., 2020; Parnaudeau et al., 2018, 2015; Yu et al., 2010). New methodological approaches will probably facilitate the development of more realistic models of the human BG, thus helping to understand the pathophysiology of BG disorders and to develop new therapeutic strategies.

## Data Availability

All data generated or analyzed during this study are included in this manuscript and are available to those researchers who request it and who agree to sing a data sharing agreement.

## Abbreviations

*PC*: Partial correlation
*GPDC*: Gaussian process regression and distance correlation
*CMIknn*: Conditional mutual information test (based on nearest-neighbor)
*M-Tal*: Motor thalamus
*IL-Tal*: Intralaminar thalamus
*MD-Tal*: Medial-dorsal thalamus
*M1*: Prim ary motor cortex
*S1*: Primary somato-sensory cortex
*Put*: Putamen
*GPe*: External globus pallidum
*STN*: Subthalamic nucleus
*GPi*: Internal globus pallidum
*SN*: Substantia nigra
*Tal*: Motor thalamus
